# Supplementary material for: Design and Synthesis of (Z)-5-(Substituted benzylidene)-3-cyclohexyl-2-thioxothiazolidin-4-one Analogues as Anti-Tyrosinase and Antioxidant Compounds: In Vitro and In Silico Insights
Source: Antioxidants (Basel). 2022 Sep 27;11(10):1918. doi: 10.3390/antiox11101918 (PMC9598926; doi:10.3390/antiox11101918)
Supplement: Supplementary file 1 [file antioxidants-11-01918-s001.zip › antioxidants-1921568-supplementary.pdf]

Supporting Information

For

Design and synthesis of (*Z*)-5-(substituted benzylidene)-3-cyclohexyl-2-thioxothiazolidin-4-one analogues as anti-tyrosinase and antioxidant compounds: *In vitro* and *in silico* insights

Jeongin Ko [1,†], Jieun Lee [1,†], Hee Jin Jung [2,†], Sultan Ullah [3], Yeongmu Jeong [1], Sojeong Hong [1], Min Kyung Kang [1], Yu Jung Park [1], YeJi Hwang [1], Dongwan Kang [4], Yujin Park [4], Pusoon Chun [5], Jin-Wook Yoo [1], Hae Young Chung [2] and Hyung Ryong Moon [1,*]

[1] Department of Manufacturing Pharmacy, College of Pharmacy, Pusan National University, Busan 46241, Korea
[2] Department of Pharmacy, College of Pharmacy, Pusan National University, Busan 46241, Korea
[3] Department of Molecular Medicine, The Scripps Research Institute, Jupiter, FL 33458, USA
[4] New Drug Development Center, Department of Medicinal Chemistry, Daegu-Gyeongbuk Medical Innovation Foundation, Daegu 41061, Korea
[5] College of Pharmacy and Inje Institute of Pharmaceutical Sciences and Research, Inje University, Gimhae 50834, Korea
* Correspondence: mhr108@pusan.ac.kr
† These authors contributed equally to this work.

Contents

S1. ^{1}H NMR spectrum of compound **1**……………………………………………………………3

S2. ^{13}C NMR spectrum of compound **1**……………………………………………………………4

S3. ^{1}H NMR spectrum of compound **2**……………………………………………………………5

S4. ^{13}C NMR spectrum of compound **2**……………………………………………………………6

S5. ^{1}H NMR spectrum of compound **3**……………………………………………………………7

S6. ^{13}C NMR spectrum of compound **3**……………………………………………………………8

S7. Proton-coupled ^{13}C NMR spectrum of compound **3**…………………………………………9

S8. ^{1}H NMR spectrum of compound **4**……………………………………………………………10

S9. ^{13}C NMR spectrum of compound **4**……………………………………………………………11

S10. ^{1}H NMR spectrum of compound **5**……………………………………………………………12

S11. ^{13}C NMR spectrum of compound **5**……………………………………………………………13

S12 ^{1}H NMR spectrum of compound **6**……………………………………………………………14

S13 ^{13}C NMR spectrum of compound **6**……………………………………………………………15

S14. ^{1}H NMR spectrum of compound **7**……………………………………………………………16

S15. ^{13}C NMR spectrum of compound **7**……………………………………………………………17

S16. ^{1}H NMR spectrum of compound **8**……………………………………………………………18

S17. ^{13}C NMR spectrum of compound **8**……………………………………………………………19

S18. ^{1}H NMR spectrum of compound **9**……………………………………………………………20

S19. ^{13}C NMR spectrum of compound **9**……………………………………………………………21

S20. Proton-coupled ^{13}C NMR spectrum of analogue **3**…………………………………………22

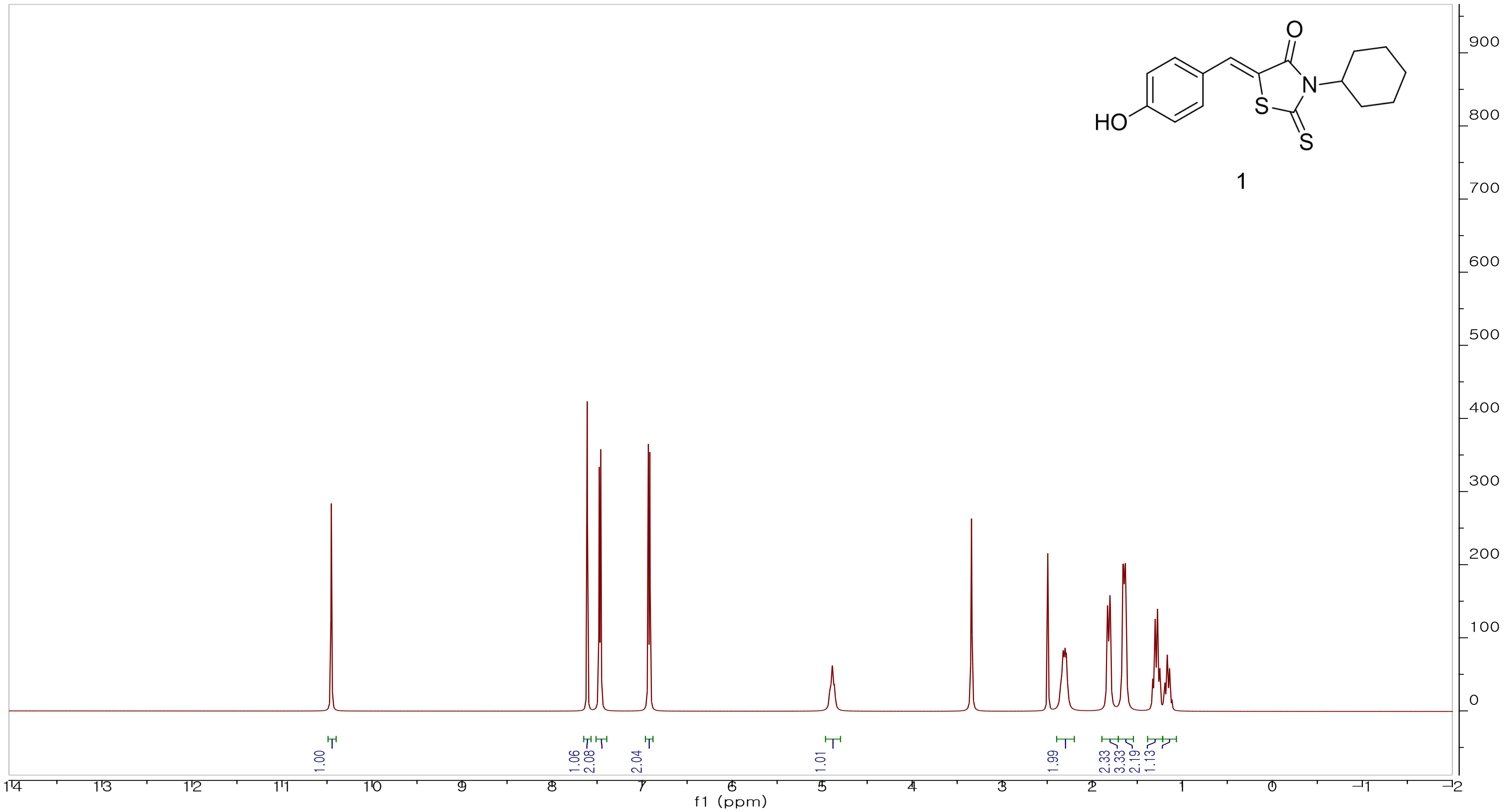

Figure S1. ^{1}H NMR spectrum of compound **1**

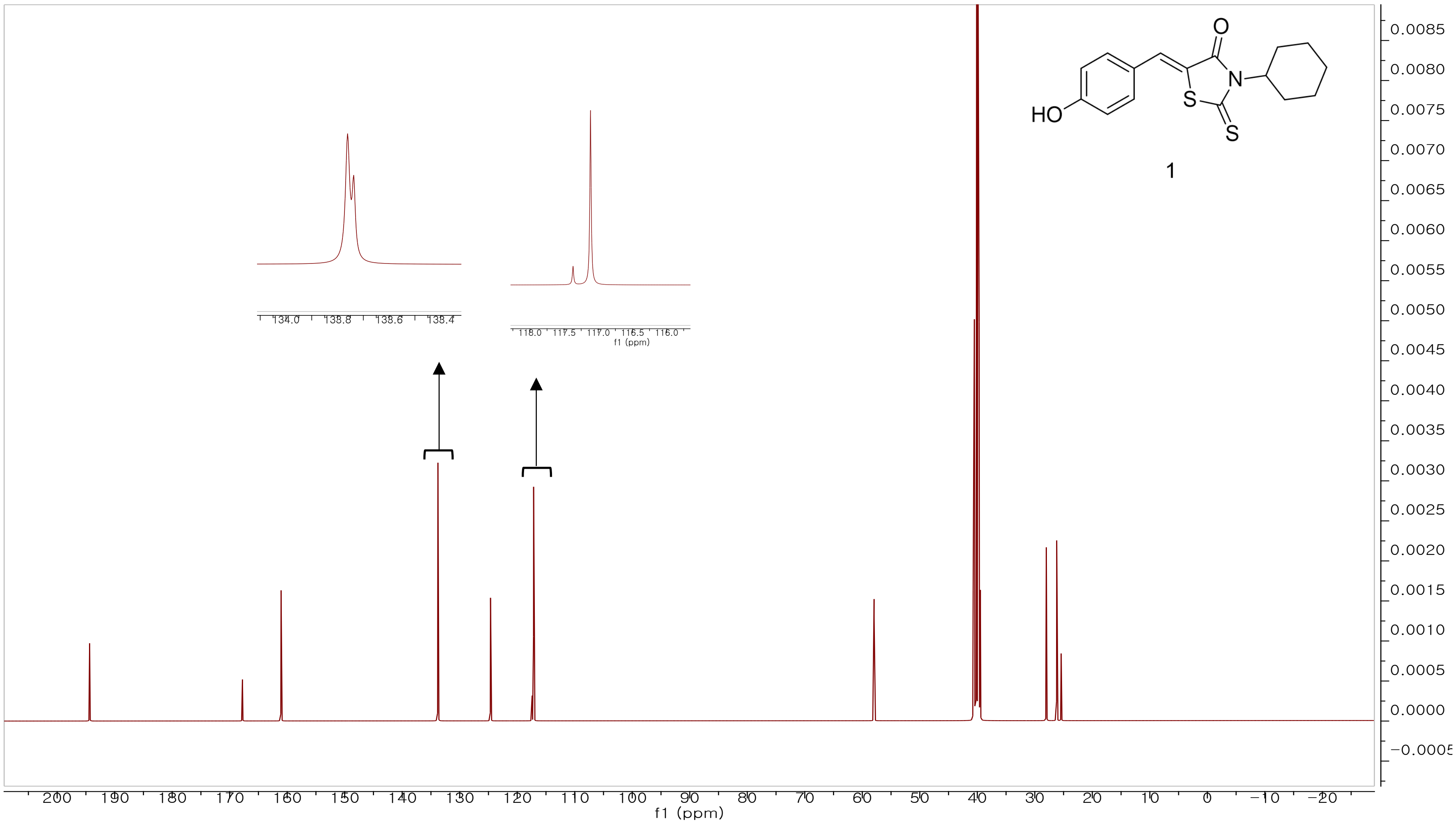

Figure S2. ^{13}C NMR spectrum of compound **1**

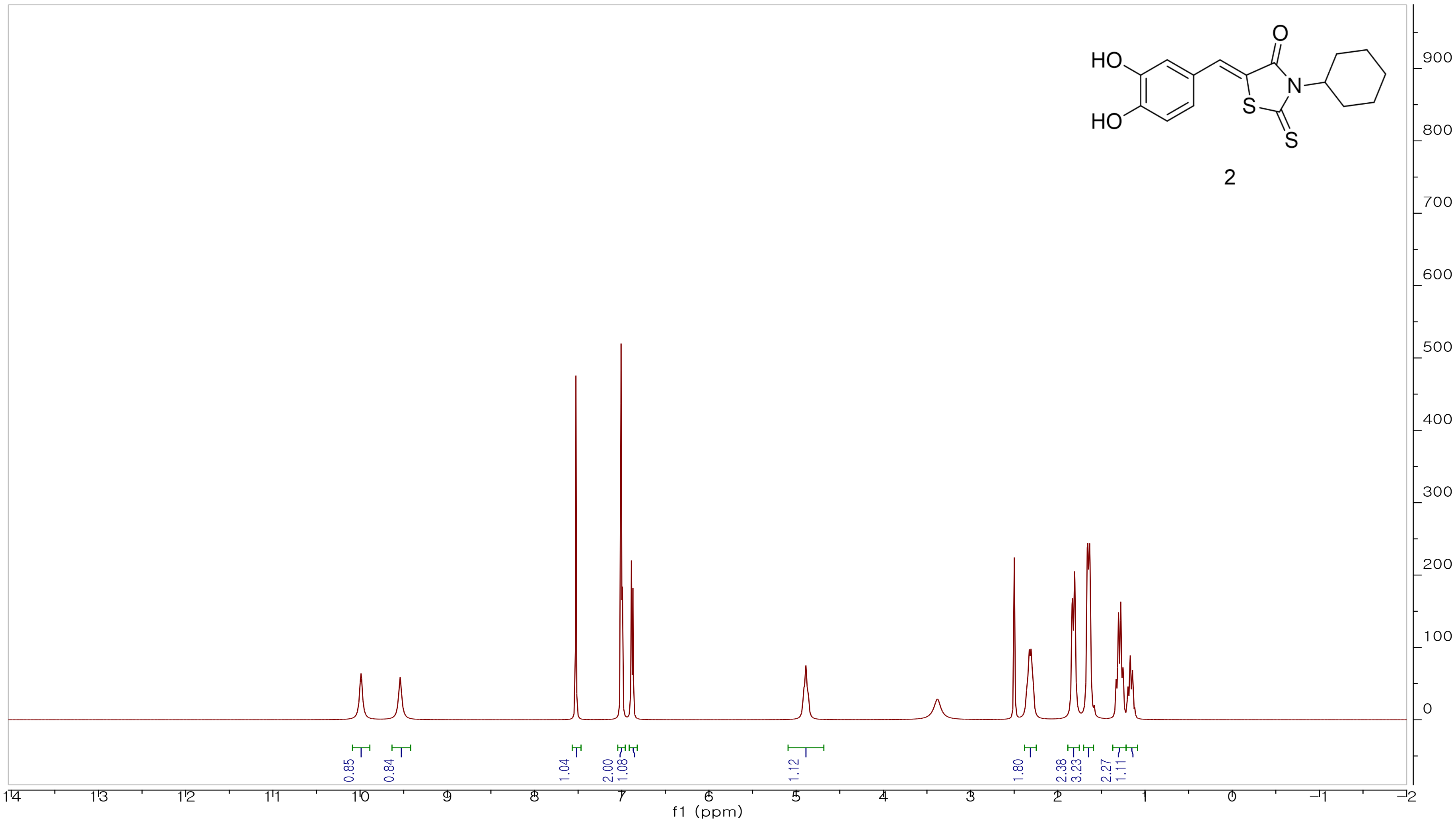

Figure S3. ^{1}H NMR spectrum of compound **2**

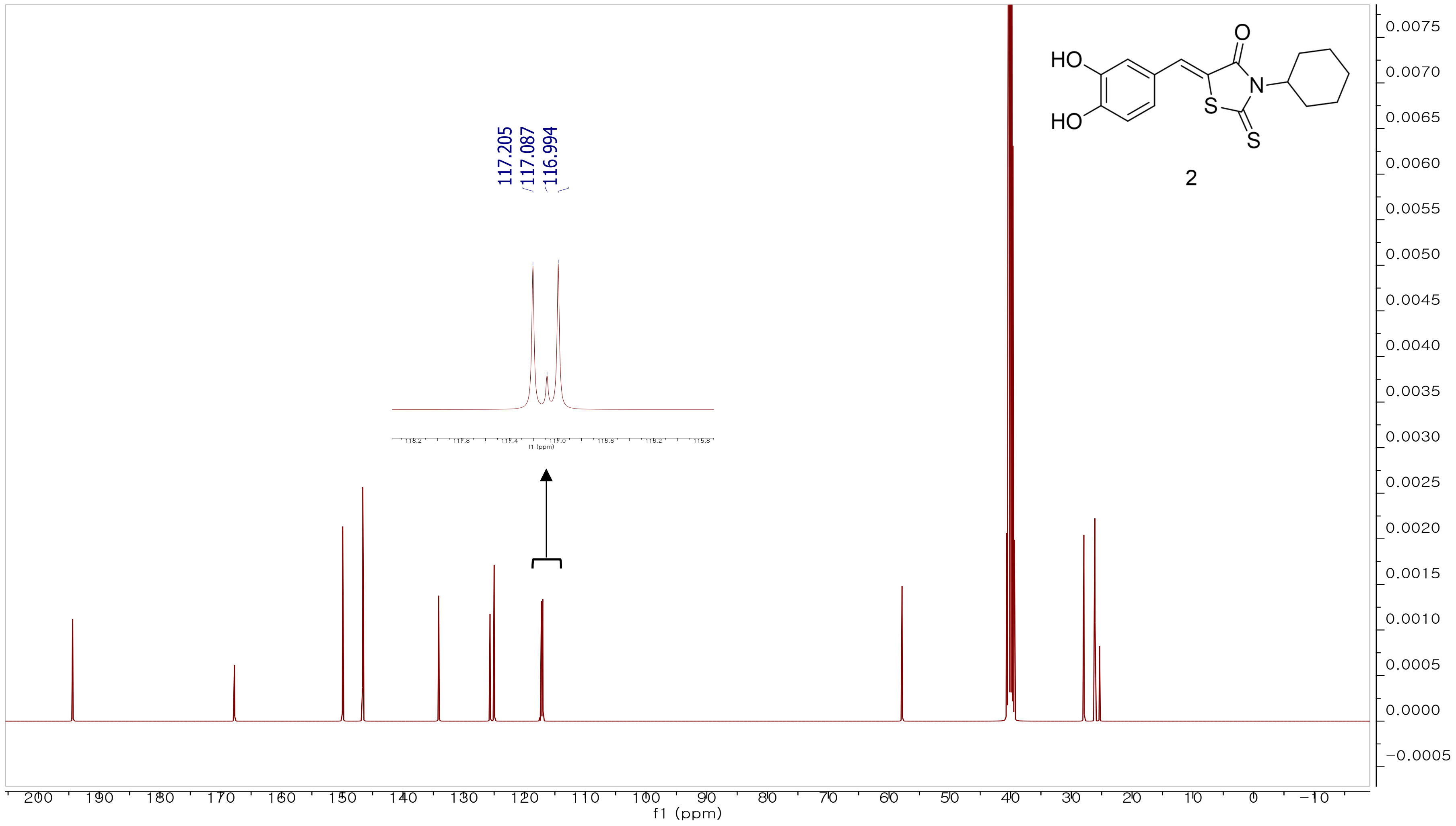

Figure S4. ^{13}C NMR spectrum of compound **2**

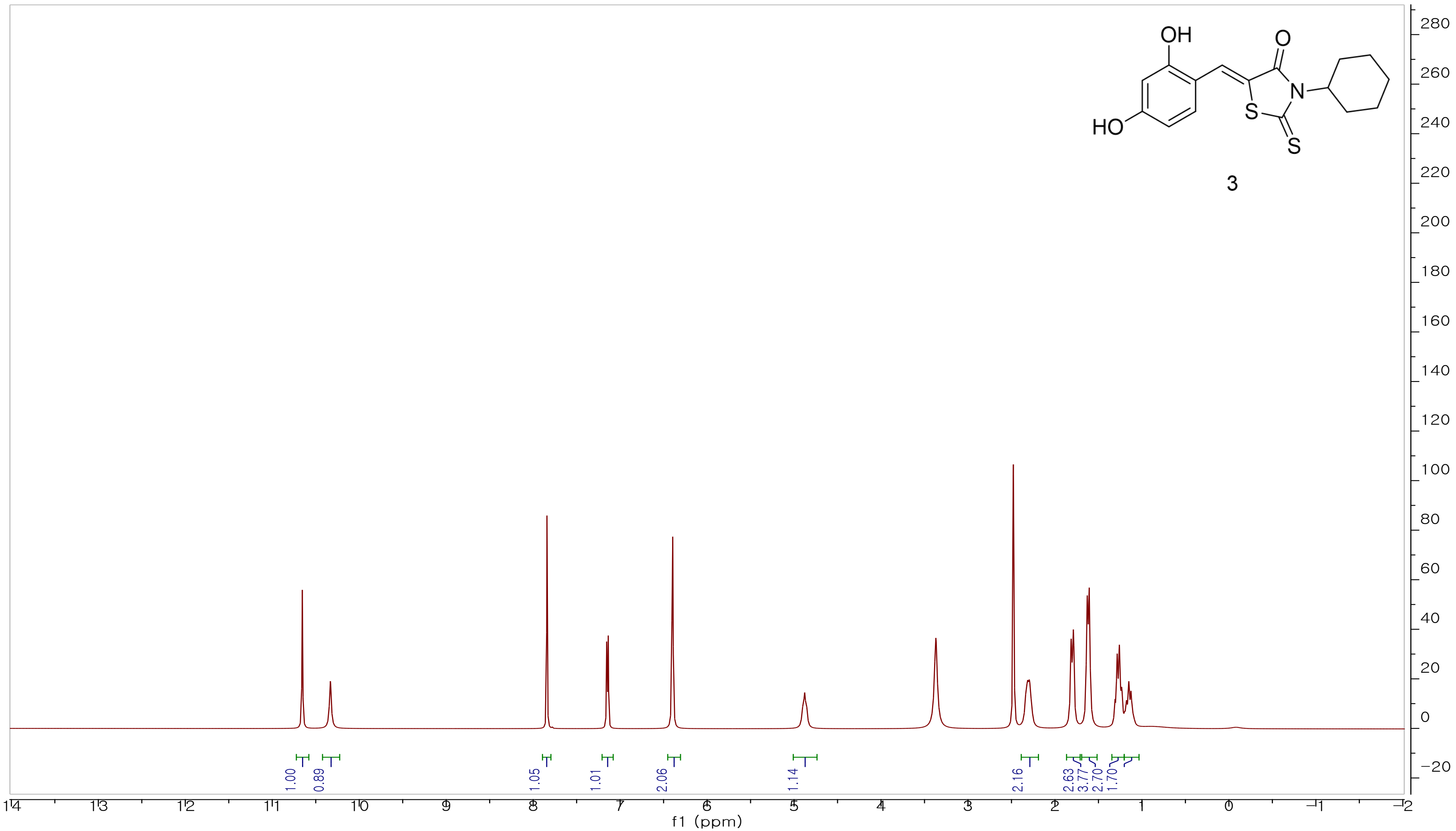

Figure S5. ^{1}H NMR spectrum of compound **3**

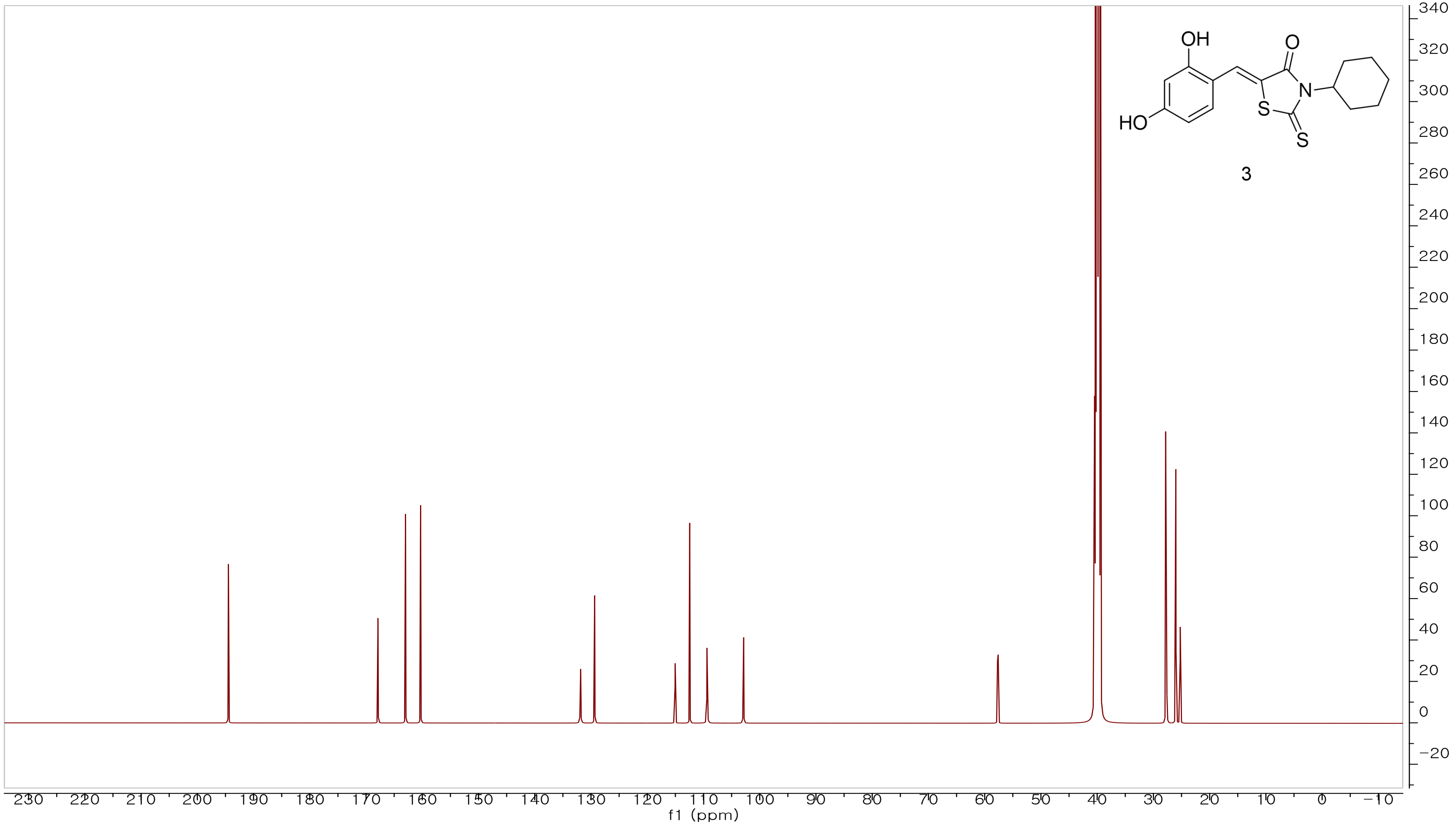

Figure S6. ^{13}C NMR spectrum of compound **3**

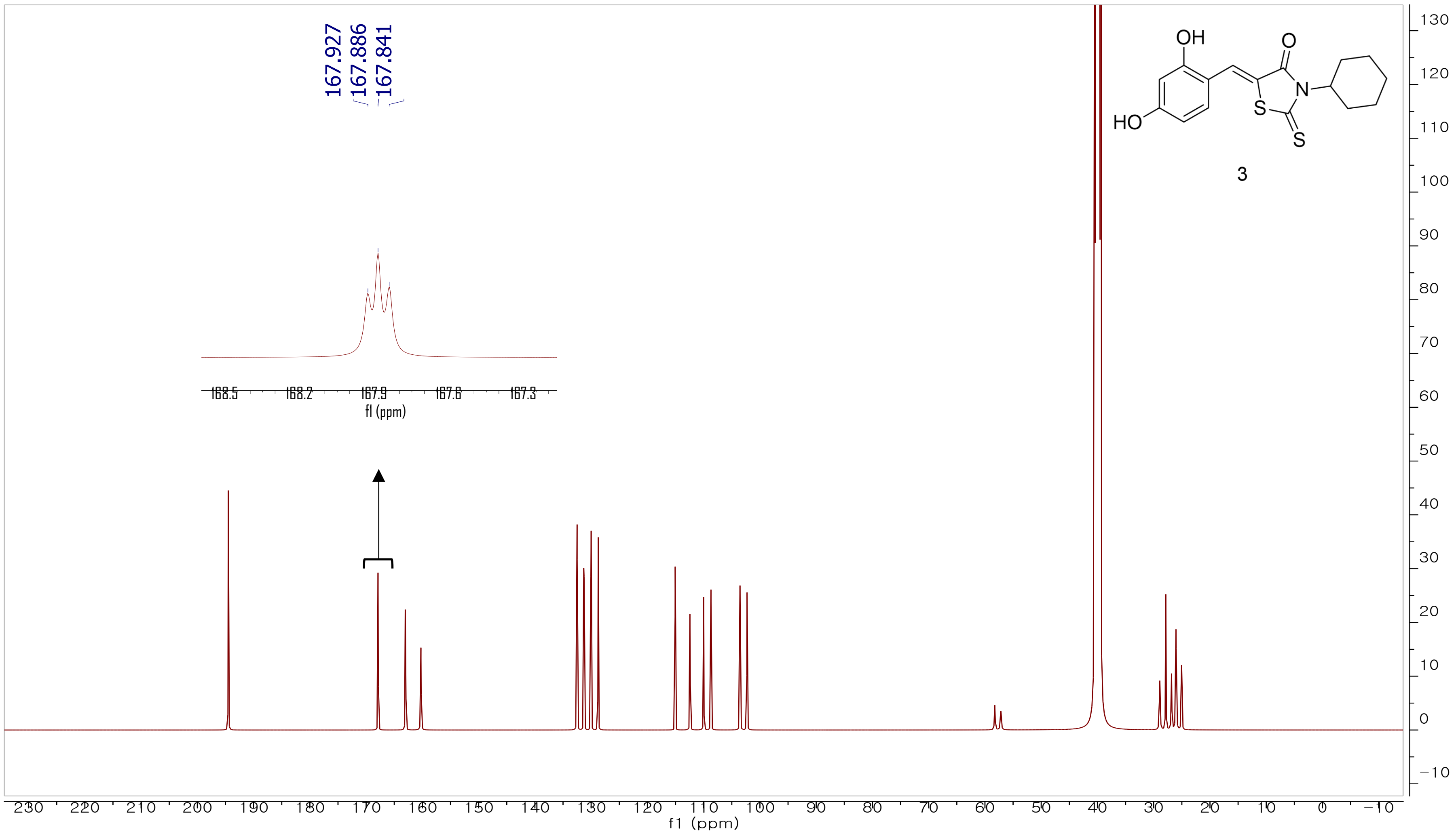

Figure S7. Proton-coupled ^{13}C NMR spectrum of compound **3**

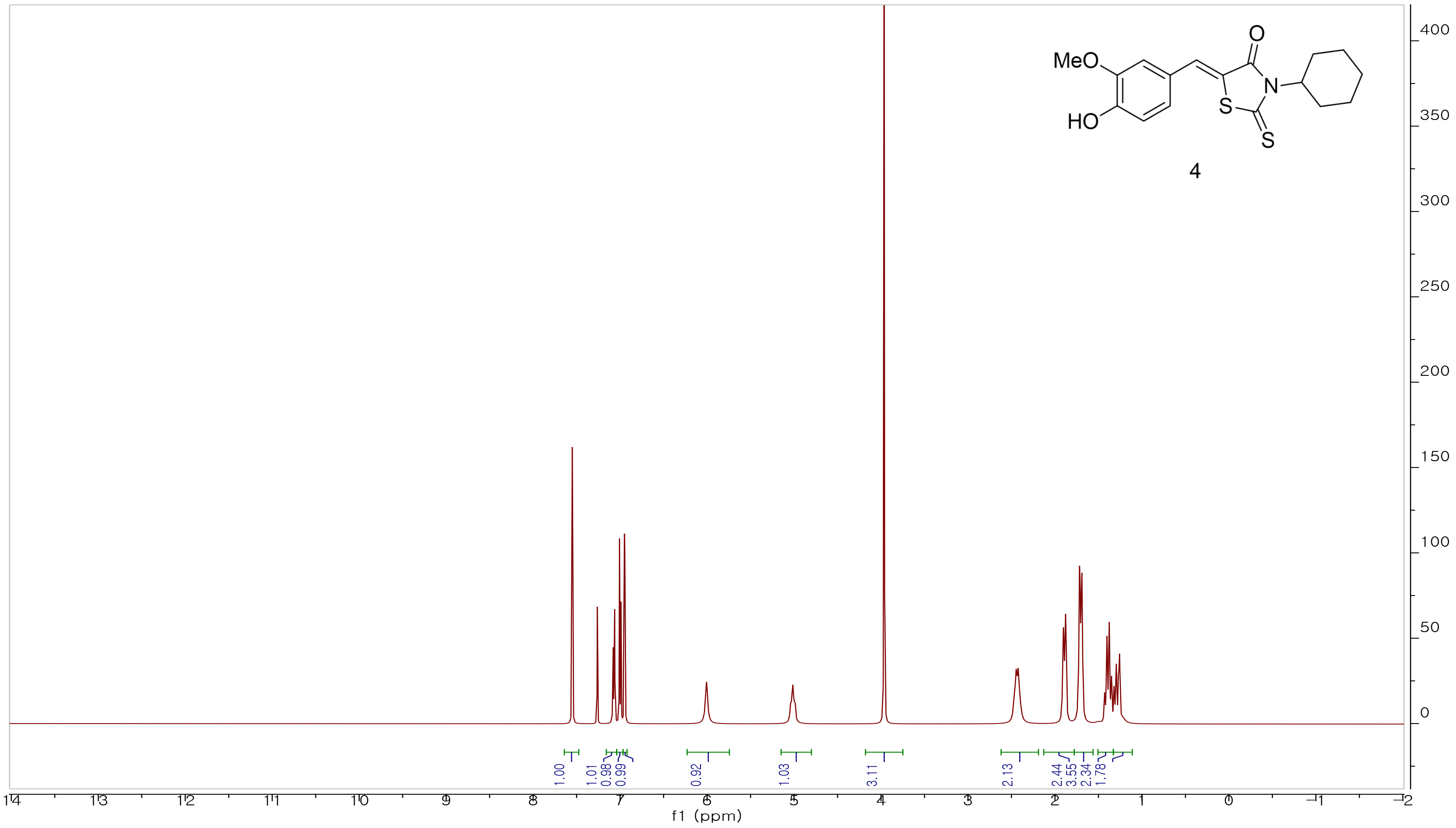

Figure S8. ^{1}H NMR spectrum of compound **4**

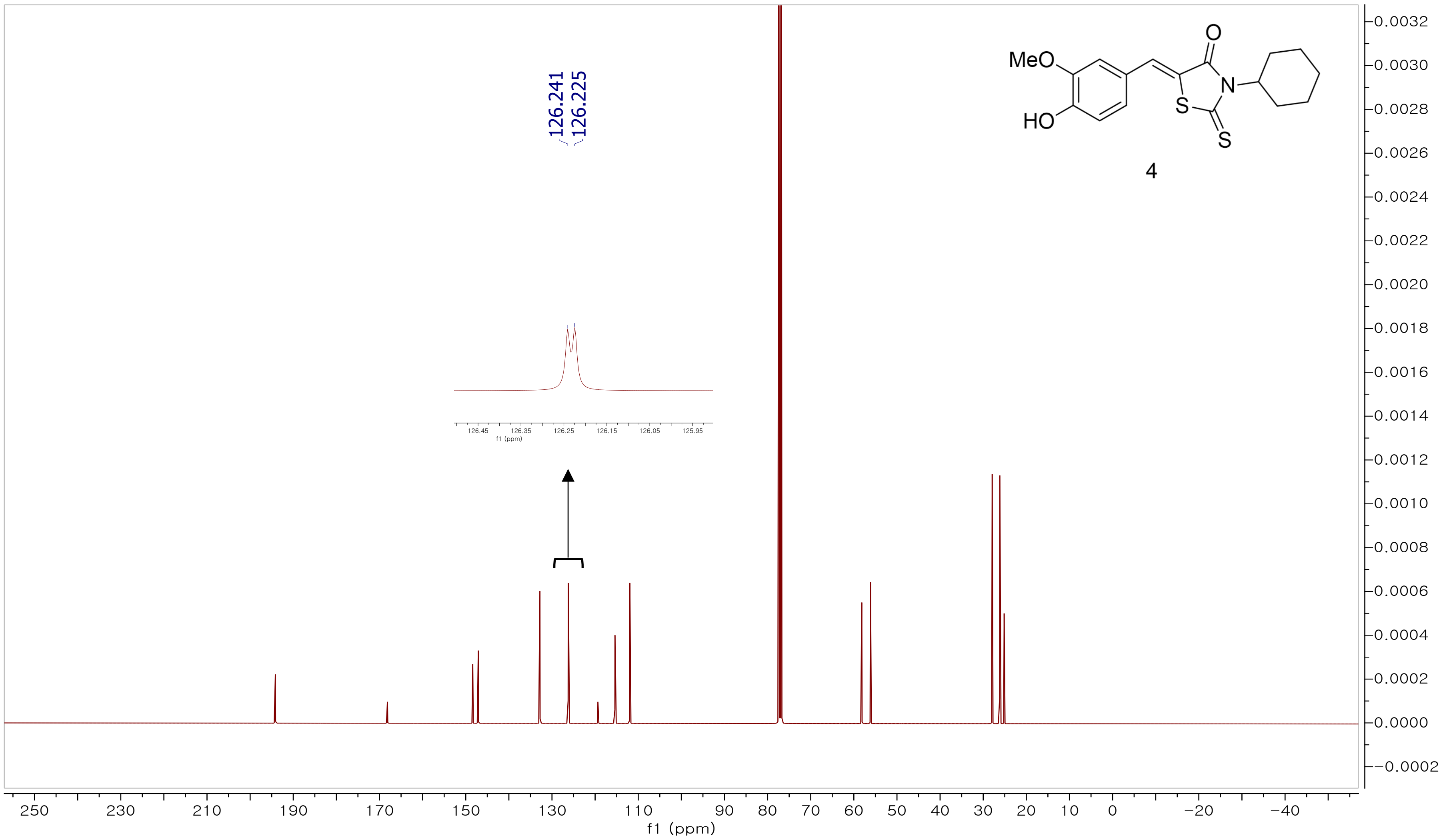

Figure S9. ^{13}C NMR spectrum of compound **4**

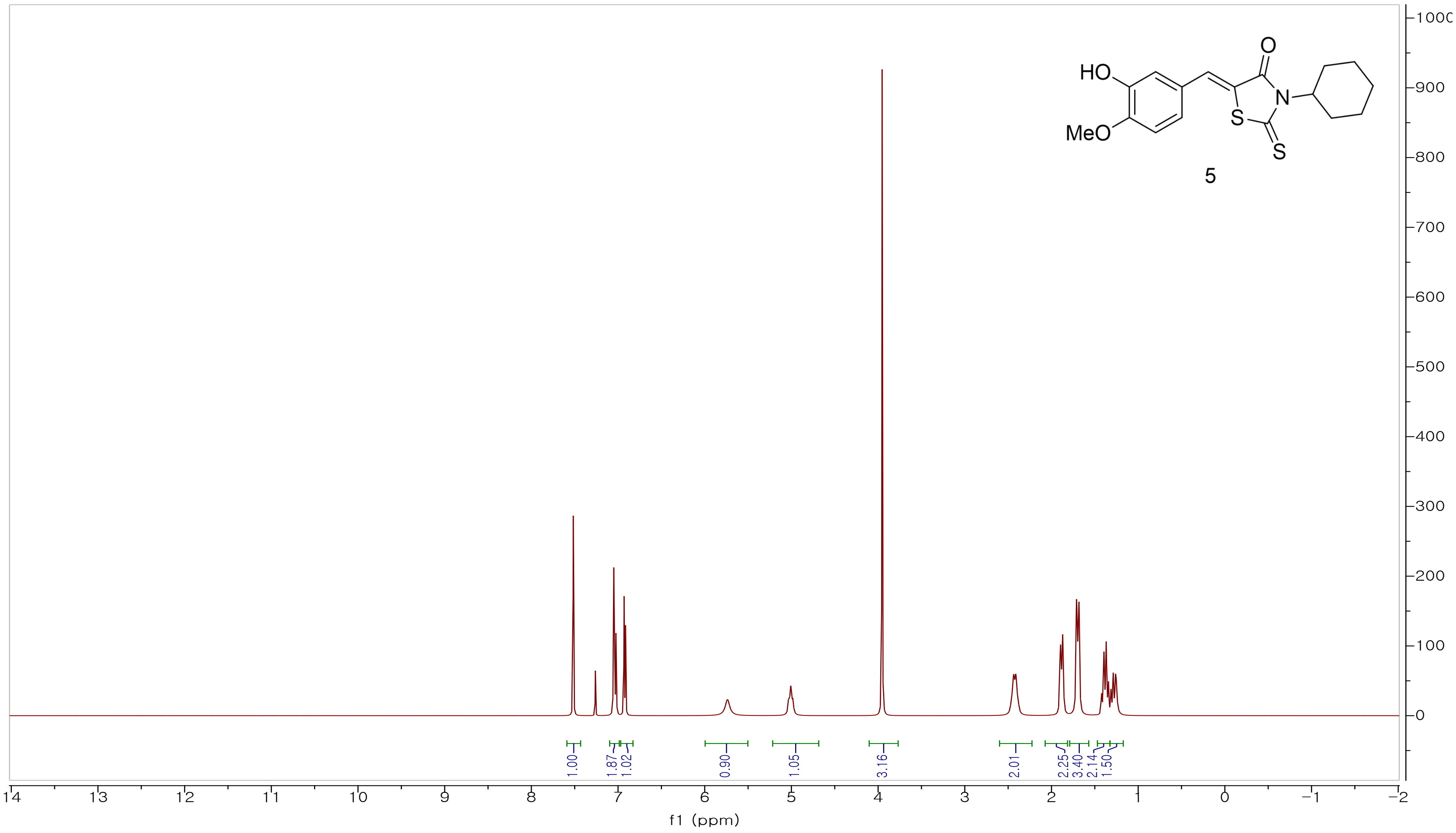

Figure S10. ¹H NMR spectrum of compound **5**

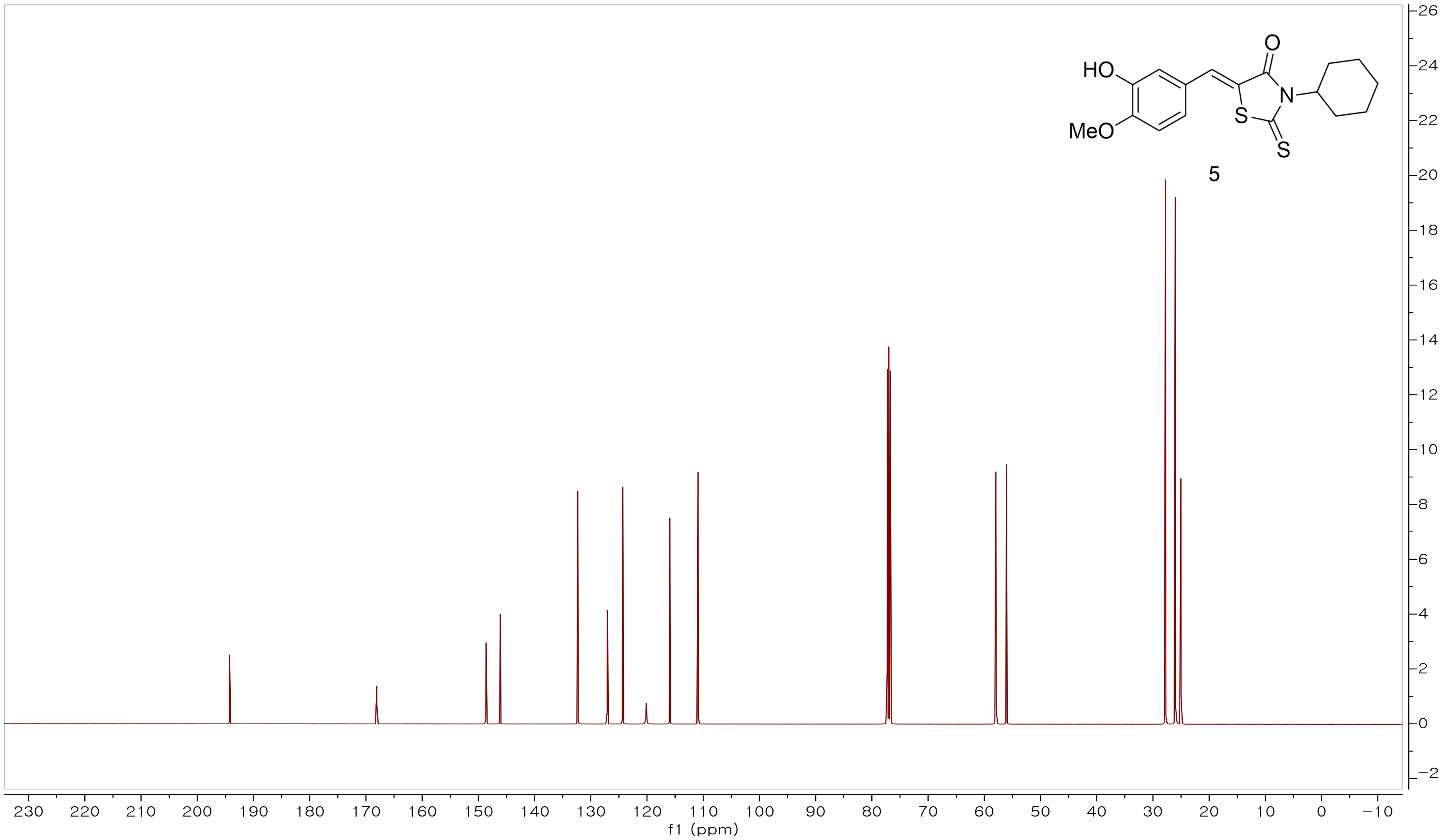

Figure S11. ^{13}C NMR spectrum of compound **5**

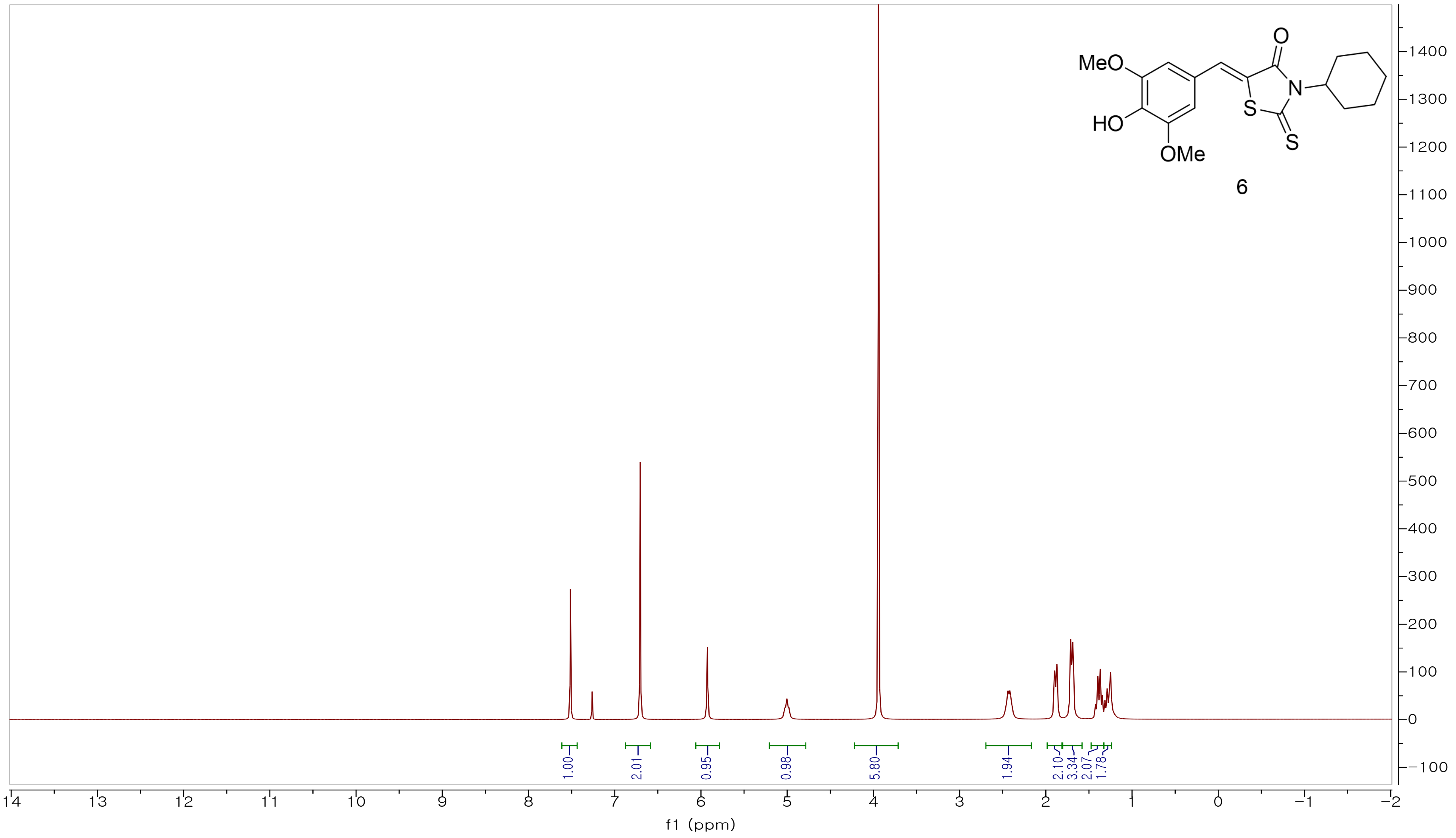

Figure S12. ^{1}H NMR spectrum of compound **6**

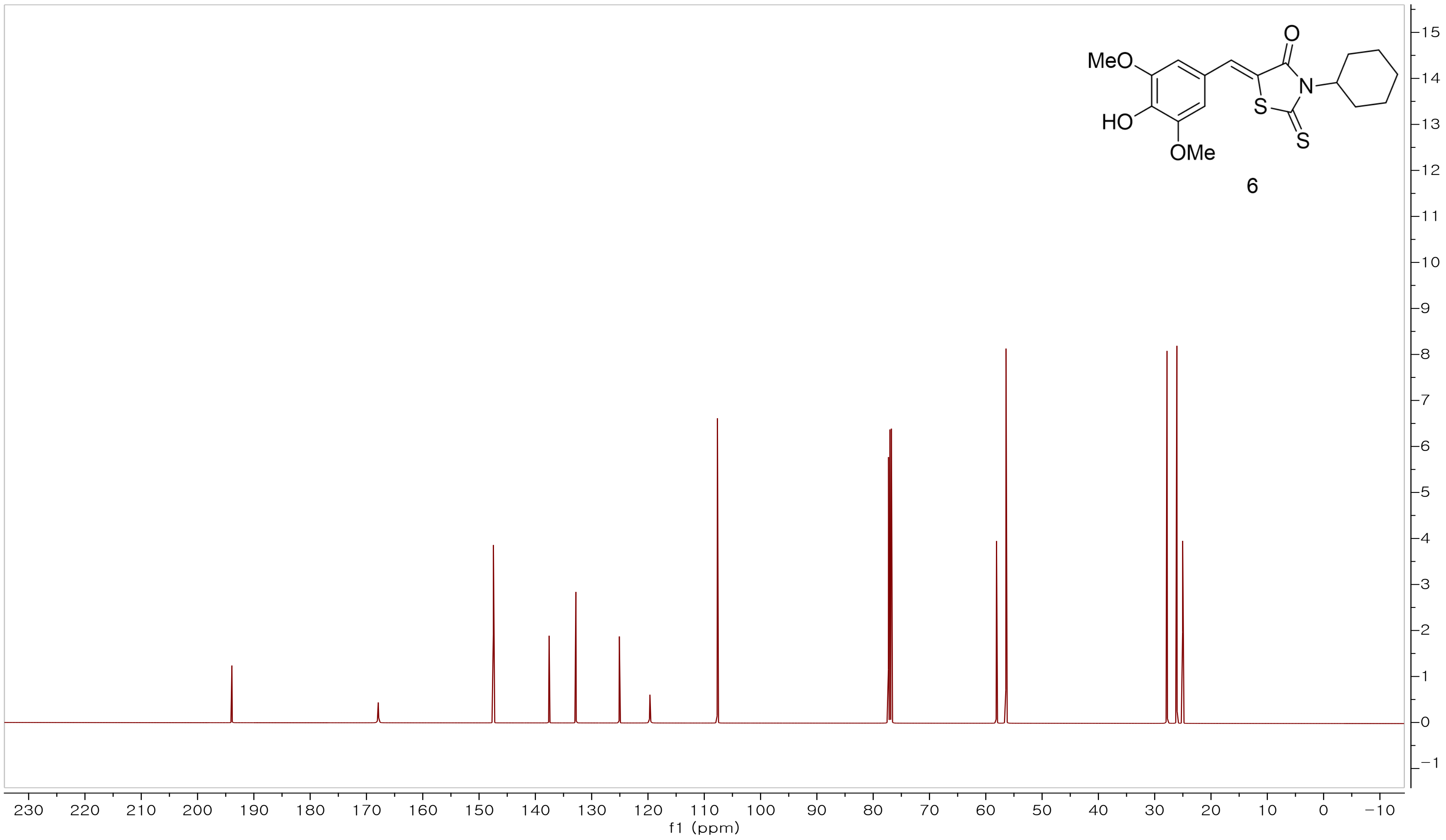

Figure S13. ^{13}C NMR spectrum of compound **6**

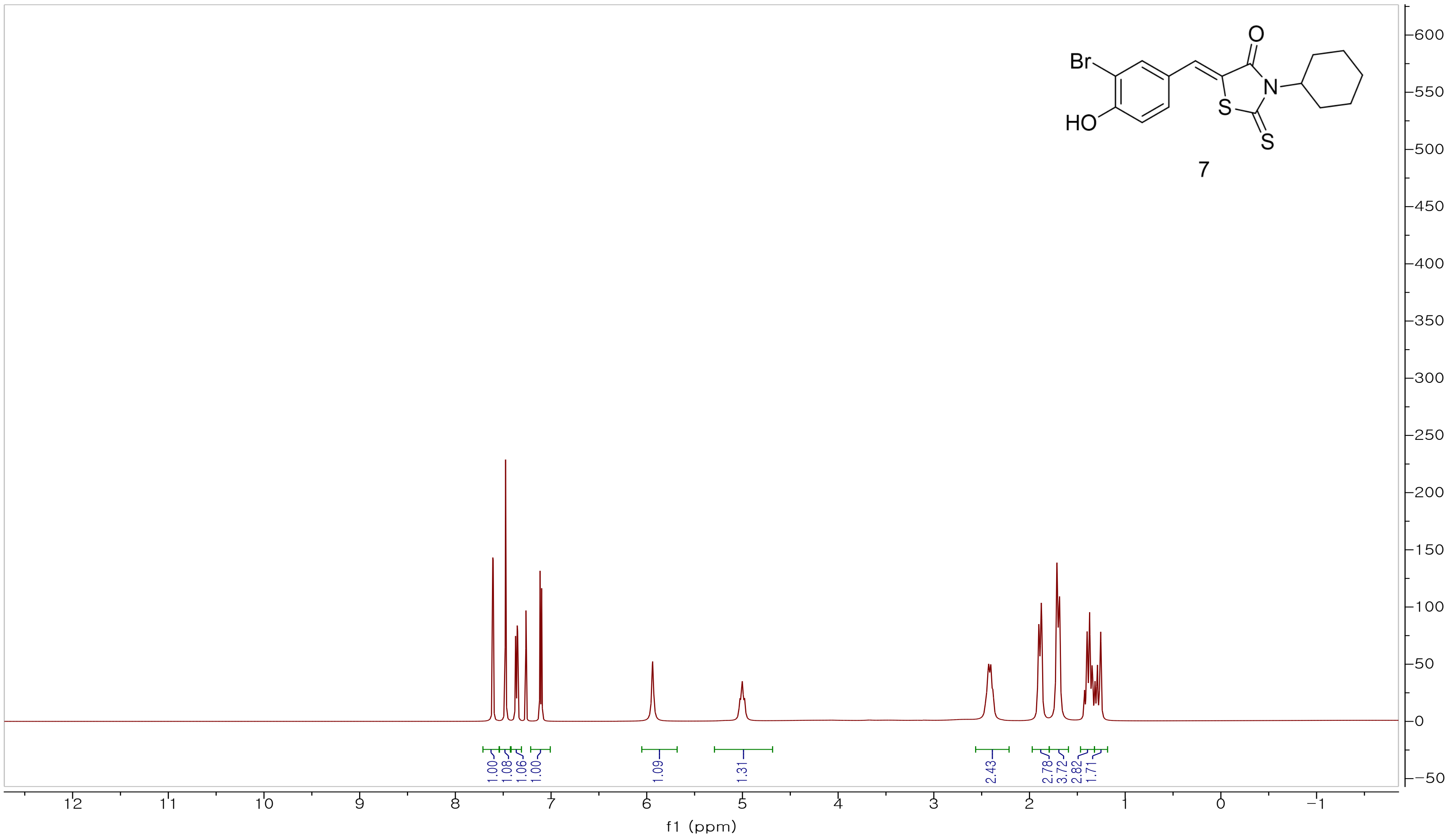

Figure S14. ^{1}H NMR spectrum of compound **7**

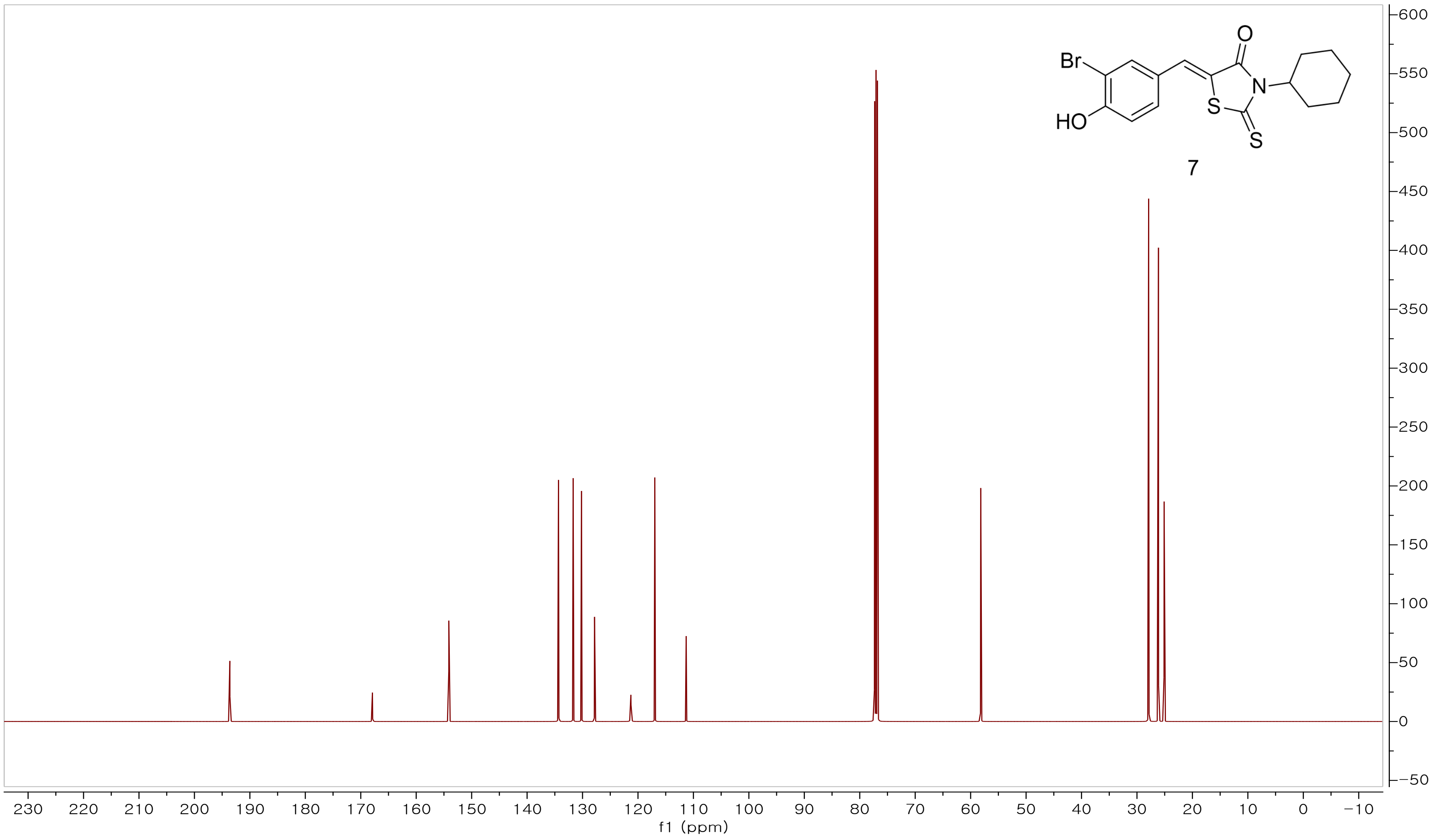

Figure S15. ^{13}C NMR spectrum of compound **7**

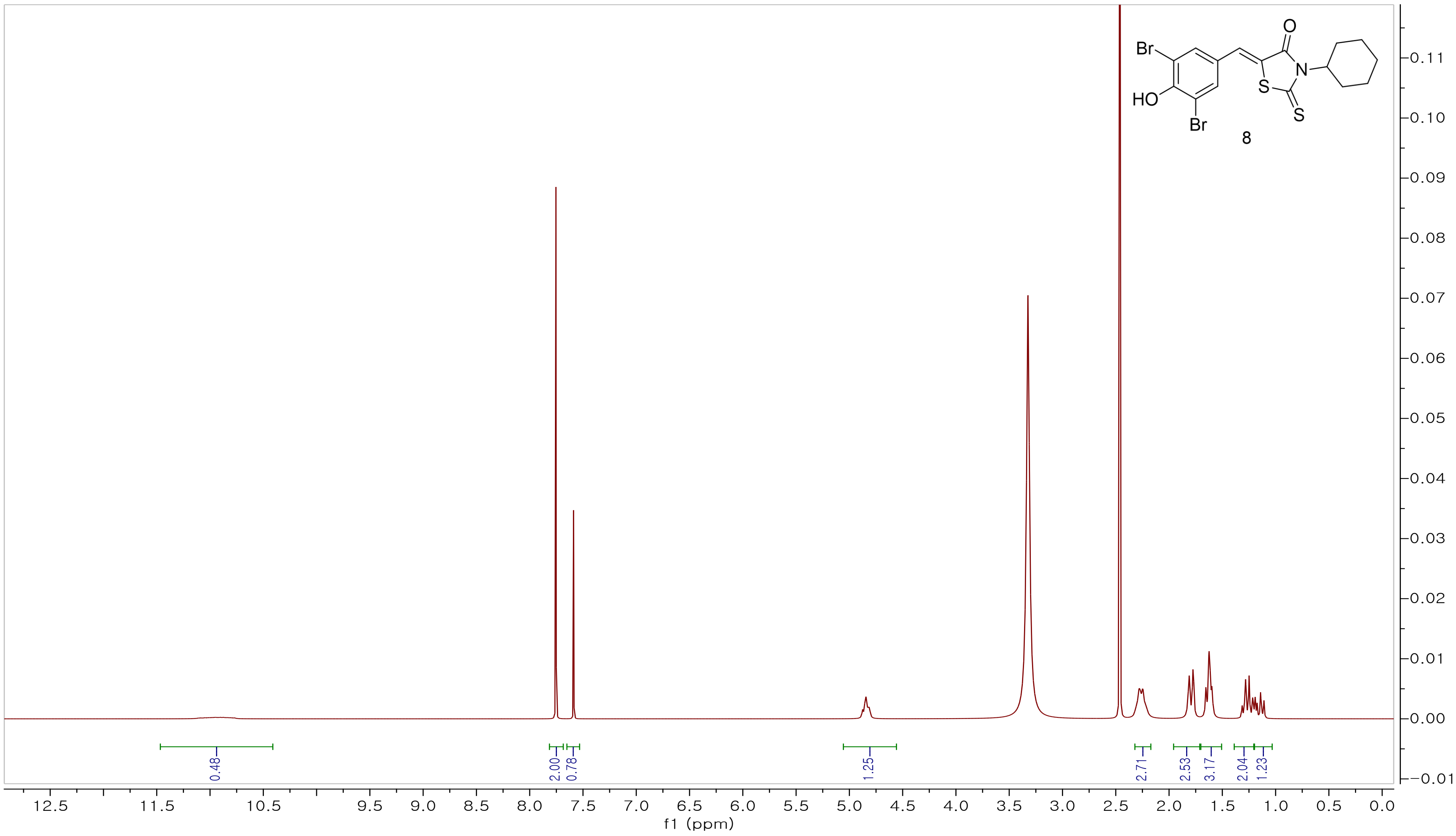

Figure S16. ^{1}H NMR spectrum of compound **8**

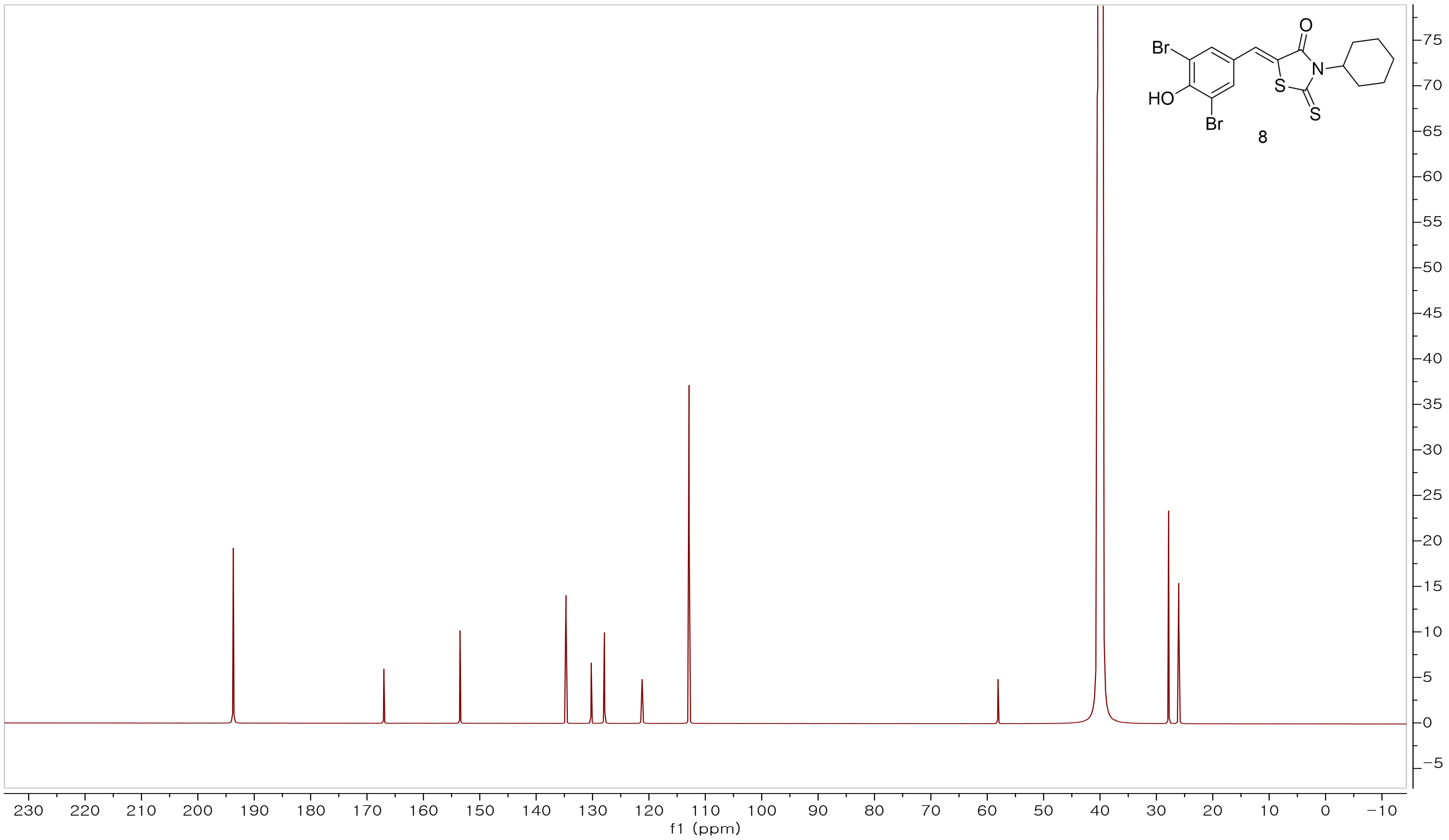

Figure S17. ^{13}C NMR spectrum of compound **8**

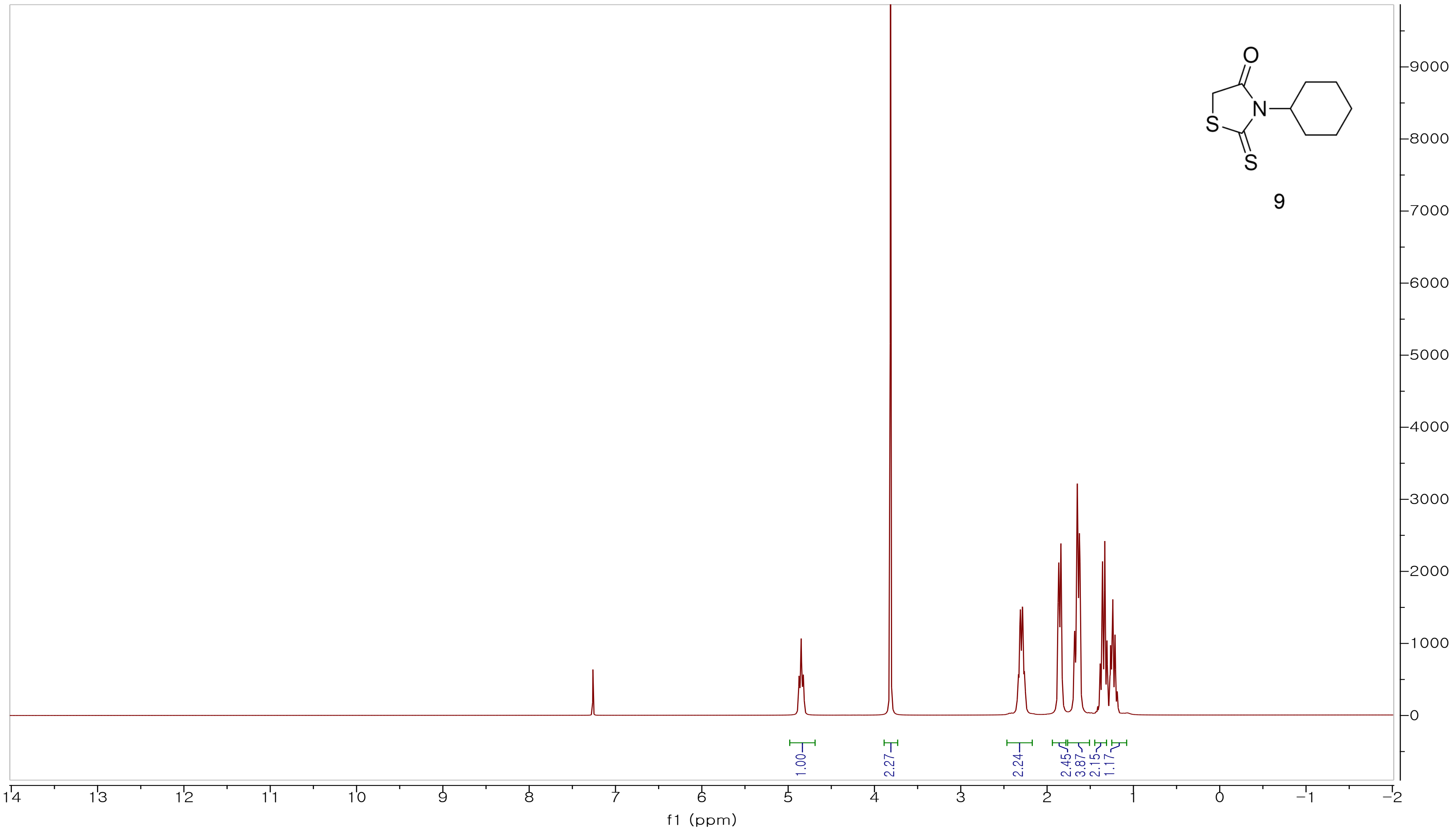

Figure S18. ^{1}H NMR spectrum of compound **9**

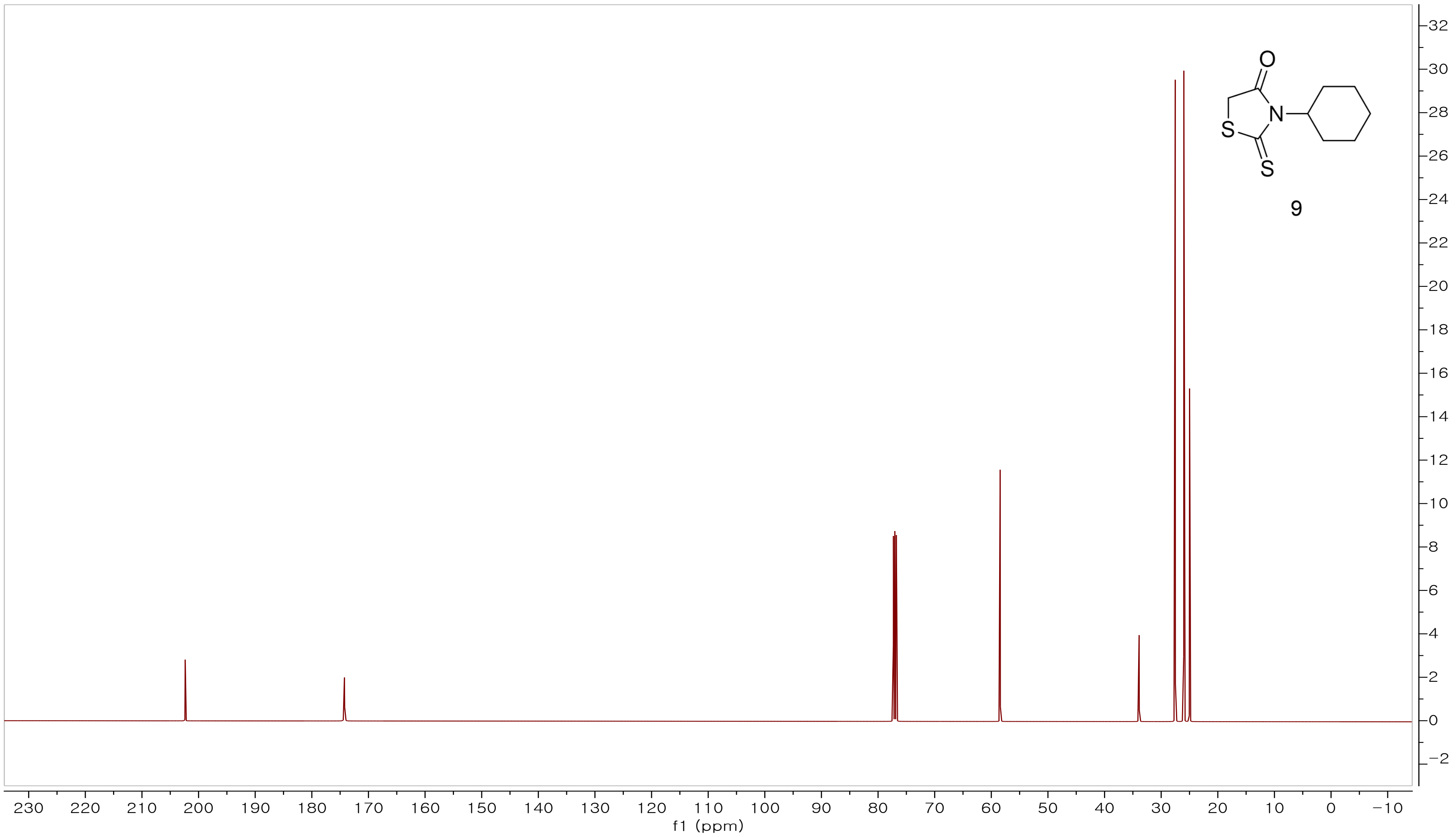

Figure S19. ^{13}C NMR spectrum of compound **9**

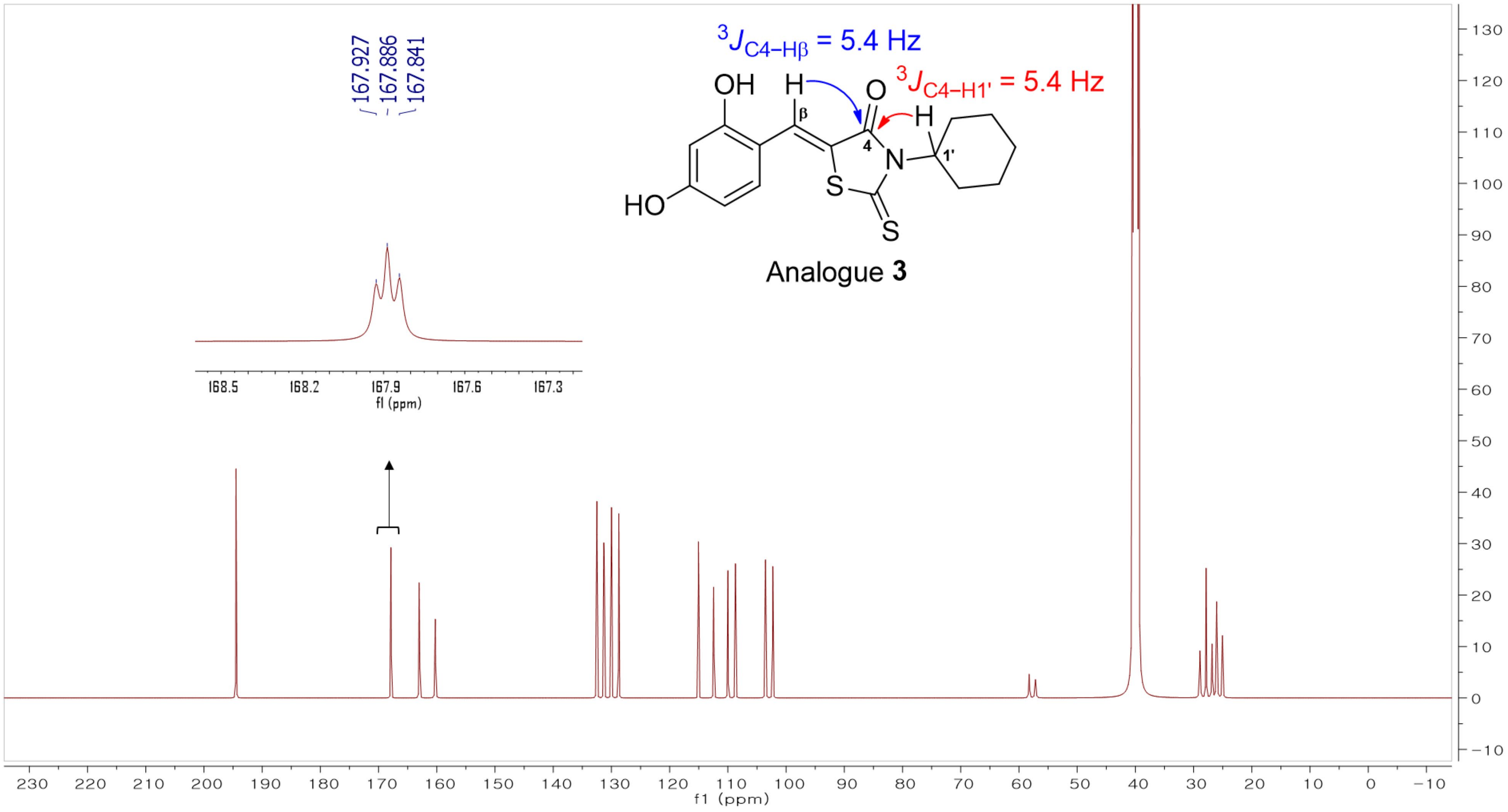

Figure S20. Proton-coupled ^{13}C NMR spectrum of analogue **3**
